# Israel and the global synthetic biology ecosystem

**DOI:** 10.1049/enb2.12027

**Published:** 2023-10-14

**Authors:** Yuval Dorfan, Aviv Zeevi, Gita Reinitz, Magi Mualem, Yosi Shacham‐Diamand

**Affiliations:** ^1^ Bioengineering HIT Holon Israel; ^2^ Alagene LTD Rehovot Israel; ^3^ Israeli Innovation Authority Jerusalem Israel; ^4^ NFX.Bio Herzliya Israel; ^5^ Shamir Research Institute Katzrin Israel; ^6^ Scojen Institute of Synthetic Biology Reichmann University Herzliya Israel

**Keywords:** automation, bio‐design, bio‐economy, biomaterials, bioprocess engineering, biosensors, genetic circuits, genertic systems, microbial engineering, synthetic biology

## Abstract

The field of synthetic biology emerged a few decades ago, following some key works of researchers in the USA, Europe, and the Far East. It reached Israel through academia and a few years later it finally got the attention of industry, venture capitals, and government authorities, especially the Israeli Innovation Authority, hoping to encourage entrepreneurs to establish startups in this field. Here we provide an overview of the activity of the field of synthetic biology in Israel, including historical notes, current strategy, prospects and developments, and further insight that are relevant to any stakeholders in the synthetic biology field.

## Graphical abstract

The field of synthetic biology emerged a few decades ago, following some key works of researchers in the USA, Europe, and the far east. It reached Israel through academia and a few years later it finally got the attention of industry, venture capitals, and government authorities, especially the Israeli innovation authority (IIA), hoping to encourage entrepreneurs to establish startups in this field. Here we provide an overview of the activity of the field of synthetic biology in Israel, including historical notes, current strategy, prospects and developments, and further insight that are relevant to any stakeholders in the synthetic biology field.

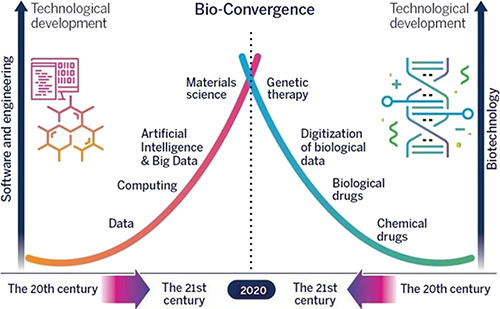

## 1 INTRODUCTION

Imagine a world where scientists can implant characteristics into living organisms to face different challenges [1]. Biological basic science provides the fundamentals and information on cell structure, proteins, living biological systems, etc. Engineering provides an application‐oriented state of mind and tools that are useful for many industrial fields. Synthetic biology (SynBio) is a new field where engineering concepts and methods are combined with those of life sciences redesigning novel biological components and systems. Synthetic biology is one of today's cutting‐edge technologies that can achieve modern industrial goals, beyond personalised health and modern agriculture. Israel has a very impressive record of being a global leader in multidisciplinary new approaches. The local ecosystem identified the broad potential of SynBio and multiple connections to Israeli strengths in science and industry. We will examine these connections and the way SynBio evolved in Israel as a case study for other countries and for other new technologies that are expected to bloom.

**FIGURE 1:**
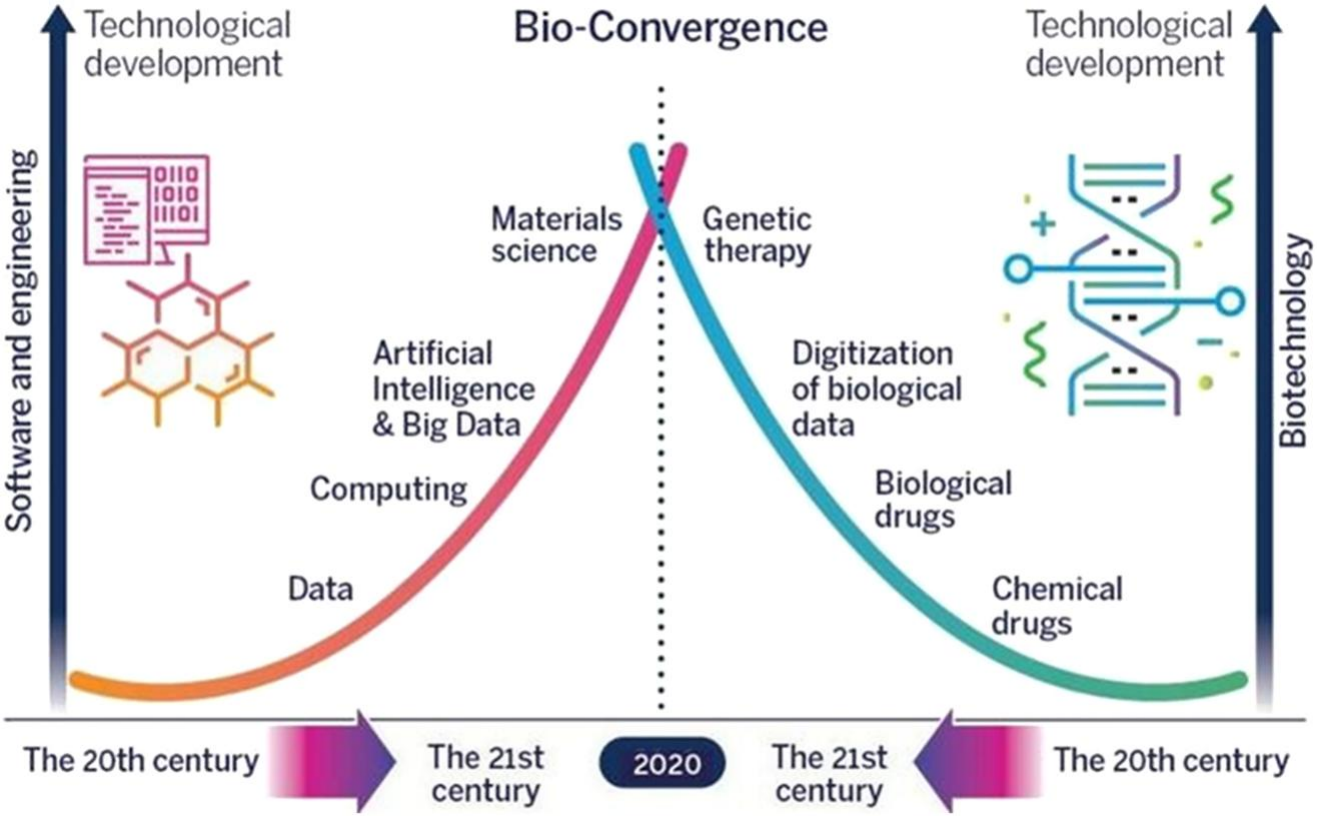
Bio‐convergence as a field that combines classic engineering with bioengineering.

From an industrial point of view, SynBio can solve problems in many fields, such as cultivated food, waste treatment, and many more. A few promising applications are discussed in Section 2. For SynBio applications, biology is part of the solution, even in cases where the problem is not biological. Using SynBio we can rebuild DNA, design gene networks or new proteins that change their behaviour, and improve cellular functions. SynBio is a promising young discipline and a very ambitious one [2] with a lot yet to be proven. Similar to its global development, in Israel it also emerged first in the academic sector, which will be discussed further in Section 3. Next, we discuss SynBio in the industrial sector supported by Israeli and international venture capital (VC) companies. A few examples of Israeli companies are described in Section 4. Next, we present the government's perspective on the SynBio revolution in Section 5. An interesting trajectory in Israel is the national bio‐convergence initiative. Bio‐convergence describes the new environment integrating tools from the fields of bioengineering (such as SynBio) and micro and nano‐scale engineering solving problems from various industrial fields such as medicine and healthcare, agriculture, food, energy, environment, defence, and homeland security (See Figure 1 below). Bio‐convergence of engineering, exact sciences, and life sciences has been considered as the basis for the next technological wave of the 21st century. For example, one of the interesting bio‐convergence demonstrations in Israel is using SynBio of microbes for landmine detection. For 75 years, humans have only identified mines using military and civilian mine detectors; A technology based on magnetic metal detectors transmitting an audio sound to the examiner's headphones. Switching from metal to plastic made landmines, a new solution was required. An Israeli group developed a bacterium sensor to remotely detect buried landmines and other explosive devices. The bacteria were genetically engineered to detect very small levels of Trinitrotoluene (TNT) and its byproduct Dinitrotoluene (DNT) expressing luminescent proteins upon exposure to explosives. Employing a combined laser/telescope optical system, the remote detection of anti‐personnel‐landmines (APLs) was demonstrated [3]. This application and many more have been developed with the growing support of the local government including the Israeli department of defence (DoD) and the IIA.

A major enabler of applying SynBio and bio‐convergence is training and education. Following the academic sector initiation, we can already identify a few academic courses in the field. However, more is needed to push the ecosystem forward. A detailed discussion about education and training from middle school to professional levels is the subject of Section 6.

## 2 SynBio APPLICATIONS: WIDE INFLUENCE ON INDUSTRIAL FIELDS WAY BEYOND HEALTH AND FOOD

SynBio is a new engineering field with various applications (T. C. [4, 5, 6]). We adopt the common definition: “redesigning organisms for useful purposes by engineering them to have new abilities.” This definition applies to any organism, and the problem solved can be taken from almost any industrial field. SynBio is expected to address global challenges in various fields such as health, security, food, agriculture, materials, chemicals, water, air pollution, soil monitoring, sustainability, space, etc.

The local ecosystem arranged a few brainstorming forums and looked for applications that have high global commercial potential and are aligned with local strengths. The decision was to focus on the applications with the highest short‐term potential. SynBio solutions can be divided into a few categories: bio‐production, bio‐actuation, and bio‐sensing. Bio‐production is about designing organisms as a factory for a certain material [7], for example, Chemical supplements, drugs, or food. This alternative way of production could be more economical and with a smaller footprint on earth than the processes designed by traditional industry based on chemical or other engineering disciplines. Bio‐production could also enable on‐site production instead of central production in big factories and transportation [8]. On‐site production can solve issues of shelf‐life and provide customers with fresh supplies that are cheaper, healthier, and independent of supply chains. Bio‐actuation refers to situations where Syn‐Bio modifies the properties of the organism or plant. For example, improving stress tolerance in plants. Bio‐sensing refers to situations where biology is used for sensing, having the advantage of specificity and sensitivity compared to conventional solutions [9]. On the other side of the spectrum, there is also a need for general alerts. Biology is then used to detect general stress. General stress (e.g., dehydration, heat shock, toxicity, etc.) relates to conditions that should be explored in detail, and their early identification is important.

## 3 ACADEMIA

Israeli academia is showing growing activity in the field of SynBio. It is supported by all the major research universities in Israel and is usually clustered at the faculties of life sciences, exact sciences, and engineering. Currently, it is led by a few researchers who run their labs and also use local interdisciplinary equipment centres such as the nano and microfabrication facilities at the main research universities: the Technion Institute of Technology (IIT), the Weizmann Institute, Bar‐Ilan University (BIU), Ben‐Gurion University (BGU) of the Negev in Beer Sheba, Tel Aviv University (TAU), the Hebrew University in Jerusalem and the Holon Institute of Technology (HIT). There is additional activity in the field of agriculture and food at the Volcani Agricultural Institute, Tel‐Hai College of Galilee, and MIGAL Galilee Research Institute Ltd. The Israeli universities are all public institutes, except Reichmann University which has been recognized in 2022 as a private university. The universities in Israel operate under the directives of the Israeli High Education Committee, however, their academic research and teaching operations are independent.

The researchers utilise their lab equipment as well as central nano‐fabrication facilities and other central service labs that are well‐equipped. The researchers in the leading universities of Israel are involved in both national and international projects; they collaborate with the leading institutes in the world and keep a strong relationship with the industry. Recently, Reichmann University established the Scogen Institute for SynBio to become a global centre for interdisciplinary research and bring together scientists, engineers, and clinicians from all over the country and the world. Finally, the Israeli government has decided to promote a national initiative of bio‐convergence, which includes SynBio. The list of researchers working on SynBio, and related topics is long and includes researchers from all the major institutes. Please find below a short list of Israeli PIs (Table 1).

**TABLE 1:** Main Israeli PIs.

#	Name	Affiliation
1	Prof. Roei Bar‐Ziv	WIS
2	Prof. Ron Milo	WIS
3	Prof. Roee Amit	Technion‐IIT
4	Prof. Ramiz Daniel	Technion‐IIT
5	Prof. Michael Levy	Technion‐IIT
6	Prof. Dan Bracha	Technion‐IIT
7	Prof. Lital Alfonta	BGU
8	Prof. Eyal Arbely	BGU
9	Dr. Miriam Amiram	BGU
10	Prof. Shimshon Belkin	HUJI
11	Dr. Lior Nissim	HUJI
12	Prof. Adi Avni	TAU
13	Prof. Tamir Tuller	TAU
14	Dr. Yuval Dorfan	HIT
15	Prof. Yosi Shacham‐Diamand	RUNI

Sustainability is an area highly influenced by SynBio. as shown in Prof. Ron Milo's lab from the life sciences discipline at the Weizmann Institute of Science (WIS). His key area of research is carbon fixation, the crucial process that supplies our food and fuel and has a huge impact on the biosphere. The lab explores the potential of using microbes to convert CO_2_ into various valuable products such as food, fuel, bioplastics, and more. By employing a combination of computational and experimental SynBio tools, the lab was able to construct a carbon fixation pathway in *Escherichia coli* [10]. This engineered strain can now utilise CO_2_ as its sole source of carbon, presenting a promising and sustainable avenue for future advancements in biotechnology. Another active lab in the field is led by Dr. Yuval Dorfan from electrical engineering at HIT. His lab is exploring plastic biodegradation, corrosion prevention, and anti‐mould probiotics.

Another important research area is application‐driven computational biology, led by Prof. Tamir Tuller from the faculty of engineering at TAU. The research in the laboratory is multidisciplinary and combines tools from disciplines such as electrical engineering, computer science, molecular biology, biophysics, and molecular evolution. His lab has developed a generic computational approach for modelling and engineering gene expression [11] and for improving the genetic stability of intracellular circuits. The technology developed in his lab has been used in various applications including vaccine development, design of oncolytic viruses, food tech, efficient antibody production, novel mRNA‐based therapies, biosensors, and more. The lab has successfully collaborated with around 15 different biotech companies (most of them from Israel) and has produced a few ‘spin‐offs’ (e.g., Synvaccine and Imagindairy). In addition, the lab frequently publishes public tools that researchers can use in the field of SynBio [12, 13].

Another growing research area in SynBio is bioengineering, led by a few PIs such as Prof. Ramiz Daniel from the Technion IIT. Prof. Daniel develops biosensors and studies the basic principles of bio‐circuit design in living systems (logic gates, analog circuit design, oscillators, and memory devices) [14]. He is involved in modelling biological systems with electronic components and also develops industrial applications. One major application is bioelectronics: biosensors for detecting chemicals in water and food [15], biosensors for detecting biomarkers in stool samples, microbial fuel cells for generating electricity, and the development of systems that integrate SynBio and electronics. Daniel's lab has recently developed several therapeutic bacterial cells for treating different diseases, such as Colorectal cancer and inflammatory bowel disease. Similarly, Dr. Yuval Dorfan at HIT studies bioengineering and takes an “application‐oriented” point of view on SynBio. The starting point is a specific industrial challenge that can be addressed by manipulating bacteria [16]. The first step is a computational design, examined by simulations [17, 18, 19]. Then, the solution is tested in the laboratory and in real‐life conditions. He works with biosensors and beneficial bacteria for green new technologies. The group is also developing new engineering techniques to influence the behaviour of bacteria. Additionally, Dr. Dorfan is one of the co‐founders of the first Israeli bio‐foundry introduced in Subsection 5.1 below. Founding Alagene is the first step in his vision to connect the local ecosystem stakeholders. Part of HIT's strategy to build strong connections with the industry is to encourage its faculty to be involved in founding and supporting local companies. Another important step in Dr. Dorfan's vision is the new M.Sc. programme for SynBio and bio‐convergence launched this summer at HIT, as described below in Subsection 6.1.

Another promising area of research belongs to medical sciences. Dr. Lior Nissim from the Hebrew University in Jerusalem is involved in the research and development of synthetic gene circuits for cancer immunotherapy [20]. Dr. Nissim develops SynBio platforms to overcome major challenges in cancer immunotherapy, including the rarity of targetable tumour‐specific antigens, tumour‐mediated immune suppression, and the cytotoxicity caused by systemic immunomodulator administration. His lab designs and optimises synthetic gene circuits that precisely identify tumour‐specific gene regulation patterns and generate the co‐expression of multiple synthetic and native immunostimulatory outputs only from within cancer cells [21]. Thus, the circuit selectively converts cancer cells into ‘Trojan horses’ that initiate potent anti‐tumour immune responses, capable of significantly reducing tumour size in vivo and prolonging mouse survival. This approach has the potential to enable powerful new immunotherapies and to study tumour immunology. Dr. Nissim is also involved in synthetic promoter engineering. Cell‐state‐specific promoters are useful for both basic and applicative research but are challenging to find. His lab developed a high‐throughput synthetic promoter engineering and screening approach that could provide compact synthetic promoters with enhanced specificity to virtually any cell state without prior gene regulation data. This platform was applied to discover promoters specific to sub‐tissues of induced pluripotent stem cells derived organoids, breast cancer, and glioma cancer stem cells.

Outdoor biosensors also comprise another major area of research in Israel. For over two and a half decades, Prof. Shimshon Belkin at the Institute of Life Sciences of the Hebrew University of Jerusalem has been implementing SynBio principles in the design, construction, and testing of whole‐cell biosensors [3]. These are live microbial cells, molecularly engineered to generate a quantifiable physical signal in response to diverse external stimuli. Different molecular circuits have been designed for the sensitive detection of a broad range of environmental pollutants, eutrophication nutrients, toxic and genotoxic compounds, or pharmaceuticals in water, soil, air, food, and body‐fluid samples. In recent years, this approach has been successfully adapted to develop a microbial‐based system for the remote detection of buried landmines and other explosive devices. Prof. Belkin also collaborated for many years with Prof. Shacham‐Diamand from TAU (currently at Reichmann University) working on integrated functional biosensors [22] integrating the microbial sensors engineered by the Belkin group into miniaturised platforms developed at the Shacham‐Diamand lab [23].

Another SynBio area of research is spanning the genetic code using artificial amino acids. At BGU, there are three groups active in this area (mentioned in Table 1). For example, Prof. Eyal Arbely's laboratory takes a multidisciplinary approach to control chemical and biological processes both in vitro and in cultured cells. The lab focuses on developing and implementing methodologies that expand the genetic code, allowing for the genetic encoding of non‐proteinogenic amino acids [24]. Specifically, reprogramming one of the stop codons and utilising an orthogonal pair of tRNA synthetase and tRNA makes it possible to incorporate non‐proteinogenic amino acids with diverse chemical groups into proteins expressed in vivo. The main objective of the Arbely lab, situated in the Department of Chemistry, is to gain a comprehensive understanding of the molecular mechanisms underlying metabolic regulation by lysine acylation. To this end, acylated lysine residues and their analogues are site‐specifically incorporated into known acylation sites, enabling a wide range of structural and functional studies of site‐specifically acylated proteins.

In summary, the academia in Israel is involved in all aspects of synthetic biology. The researchers are involved in both basic and applied sciences. They are in close contact with the industry, mainly under the support of the IIA and the Ministry of Innovation, Science and Technology in Israel.

## 4 INDUSTRY

Israel is regarded for its ingenuity, problem‐solving capabilities, outside‐the‐box thinking, risk‐taking, lower budgets, and shorter timelines compared to many other ecosystems. Israel leads innovation in many fields, from irrigation and agriculture to cell therapy and Car‐T engineering. In the world of SynBio, Israel has punched above its weight in multiple categories, from academic grants to cultured meat and alternative protein production. Increasingly, you can find Israeli companies participating in globally recognized accelerator programs such as IndieBio and YCombinator. More VC interest is turned towards Israel, noting innovation, talent, and more accessible valuations relative to ecosystems such as Silicon Valley.

The industry sectors in Israel have started showing a growing interest in the field of SynBio in the last 3–4 years. Interest in SynBio began with traditional biotech applications such as drug discovery, medical therapeutics, and diagnostics and continued growth into additional sectors ranging from materials to cultured meat and alternative proteins. Examples include Evogene, BiomX, and Compugen. A few recent examples of key SynBio industries in Israel are mentioned below.

**Bio‐production:** Alternative ways to manufacture molecules and special materials have a wide range of implications, including economy, sustainability, and molecules or materials that cannot be produced using classical engineering techniques. For example, **SEEVIX**, one of the first Israeli SynBio companies, designs DNA that replicates the natural process of spider silk creation by inducing the fibre's spontaneous self‐assembly [2]. This critical step turns spider silk proteins into spider silk nanofibers, endowing them with spider silk's superior natural characteristics.

**Healthcare and Medical Research:** SynBio is being employed in various areas of medical research, including gene therapies, regenerative medicine, and personalised medicine. Additional examples of healthcare and medical companies in Israel include:

**Abintus** is developing novel, off‐the‐shelf genetic medicines that engineer cells directly inside the body to improve patient outcomes and access. **MeMed** develops innovative diagnostic solutions that combine host immune response monitoring and machine learning algorithms. Their platform utilises SynBio‐based biomarkers to accurately distinguish between viral and bacterial infections. **CollPlant** is a regenerative medicine company that specialises in the development and manufacturing of recombinant human collagen and other bio‐inks for 3D bioprinting. **Kadimastem** is a biotechnology company that focuses on developing cell therapies for neurodegenerative diseases, such as ALS and Parkinson's disease. They use SynBio approaches to generate functional human neural cells for transplantation.

**AgriTech and Crop Sciences:** Israel is known for its advanced agricultural technologies. Recently, Israeli R&D institutes and companies have applied SynBio to enhance crop traits, improve agricultural practices, and develop sustainable farming solutions. Companies like Kaiima Bio‐Agritech and FuturaGene (now part of Suzano) are working in this area.

**Biofuel and Renewable Energy:** SynBio is being used to develop biofuels and other sustainable energy solutions. Companies such as H2 Energy Now and Global Bioenergies are focused on leveraging synthetic biology for the production of biofuels. A new consortium, called Bio‐Plast was recently approved by the IIA. It will be coordinated by Carmel Dolphins and will include relevant companies that are recruiting this technology for several applications. For example, Tama's Research and Development Department is working with both agricultural plastics and machinery, giving Tama a unique opportunity to use the technology to develop biodegradable agricultural baling solutions.

**Industrial Biotechnology:** Israeli companies are applying SynBio to create novel enzymes, bio‐based materials, and bio‐manufacturing processes. Companies like **Bioblast Pharma** and **Bonus BioGroup** are actively involved in this sector.

Another essential part of the industry is lab service providers. There are local companies already assisting classic biotech and medical companies for a few decades, such as Hylabs and DarenLabs. Recently, Alagene was founded as well, focussing on synthetic biology services. More details about Alagene can be found in the Government Section (5) since the idea to ramp up such a unique infrastructure was pushed by the IIA and a few other stakeholders from the local ecosystem.

A few years after the founding of the first SynBio companies in Israel local VC companies started to ramp up. One of the first VCs focused on SynBio, Tech‐bio, was founded by Dr. Omri Amirav Drori and Gita Reinitz in 2018 and focuses on pre‐seed investments in Israel and the US. Since then, VC's interest in SynBio has grown, and they have added this vertical to their investment thesis, including NFX, Viola Ventures, MoreVC, aMoon, Deep Insight, Meron Capital, Aleph VC, A16Z (Andreesen Horowitz), Entree Capital, Team8, Arkin Holdings, Lionbird, Innovation Endeavours, Sapir Ventures, and many more.

Techbio was a micro VC founded in 2018 with the mission to lead pre‐seed rounds in startups focussing on the intersection of tech and bio in Israel and the US. The founding investors, Omri Amirav Drori and Gita Reinitz later joined NFX, a San Francisco‐based generalist fund, to start NFXBio, leading seed rounds around the world in biology platforms, with a strong emphasis on Israeli startups. The thesis in 2018 was that TechBio was ripe with opportunities that were undervalued and underfunded at hyper early stages, where scientist founders were still finding the language and tools to build companies around the powerful technologies that they had developed, usually in the confines of academia.

These companies were enabled by multiple technological revolutions, namely, the ability to read, write and edit DNA, RNA, and proteins; the digitisation of data; and the transition from manual, slow, and error‐prone work, to high throughput automation at scale. With these technologies fuelling the tech‐bio revolution, some companies in the biological sphere started to reduce their time to market and their capital expenditure to be more in line with classic tech companies than with traditional biotech companies.

The Israeli ecosystem has not yet peaked in value and is still in the log phase of its growth. In the coming years, expect to see more academic spinouts, more talent returning from top institutions around the world, and more companies that have been in early‐stage mode, beginning to scale and reach global markets. We expect the Israeli ecosystem to continue to grow stronger, attracting capital investment and leading the way in R&D in SynBio across many verticals.

## 5 GOVERNMENT INVOLVEMENT

The Israeli government started to show growing interest in the field of SynBio for both economic and defence reasons. The Israeli DoD started to invest small amounts in specific applications and then the IIA joined with specific academic and industrial projects' support and infrastructure initiation. After a year's extensive research, the IIA identified the field of SynBio as an innovative infrastructural field based on broad and groundbreaking multidisciplinary knowledge in academia that enables the advancement of the bio‐convergence industry in Israel and the development of new companies in the field. In light of this, it was decided that the timing was right to build a team of experts to define the required activity and guidelines for establishing SynBio infrastructure in Israel. SynBio is one of the core areas of the bio‐convergence strategy and encompasses many approaches, methodologies, and disciplines focussing on engineering, computing, and biology. This field focuses on providing solutions to complex problems that require multidisciplinary knowledge by designing and building devices and biological systems for purposes useful to the needs of humans and society. Several strategic partners have expressed interest in it, including Ginkgo Bioworks, a pioneer in SynBio in the US, and other companies such as Elbit and GFI Israel. The equipment that will form the basis of the company's service provision will be divided between academia and industry, including a module for performing various syntheses and optimising expression systems, calibrating methods, cleaning and characterising products, and a functional analysis module, scanning metabolic pathways, protein production, intracellular interactions, cell imaging and more, all of which will be located in a service‐providing industry based on existing equipment and new equipment that will be purchased.

One of the industrial projects the IIA has supported was developed by Biomax, which develops both natural and engineered phage cocktails designed to target and destroy harmful bacteria in chronic diseases, such as cystic fibrosis, inflammatory bowel disease, atopic dermatitis, and colorectal cancer.

The subsections below are about specific governmental initiatives. The first one is about the first Israeli bio‐foundry, Alagene. The second subsection is dedicated to specific applications the government offices decided to support. The last subsection describes a few consortia that get together various local stakeholders to collaborate on a long‐term vision of a specific technology or application.

### 5.1 Alagene, the Israeli bio‐foundry: Infrastructure build‐up

In the past few years, a few Israeli entrepreneurs have pushed new applications, however, most have faced market barriers. To address those barriers, the IIA, together with other Israeli stakeholders from the government, industry, VCs, and academia, began planning how to close those gaps. One option was using a unique local infrastructure facility, which will serve as an enabler for the translation of ideas to the marketplace. The IIA had announced a call for proposals for a new company that would serve as the first Israeli Bio‐Foundry. The IIA offered 55% of 40M NIS for any group that can develop a professional service vision with internal financial capabilities in a short time frame of two to 3 years. The winning team was a unique combination of a classical biotech service company, Hylabs Ltd., Reichman University, and VC investors led by Michael Eisenberg. The new company, Alagene, started officially in March 2022 and is already operational, serving its first customers. This facility is only one example out of many initiatives of the Israeli government to push the local ecosystem forward.

**FIGURE 2:**
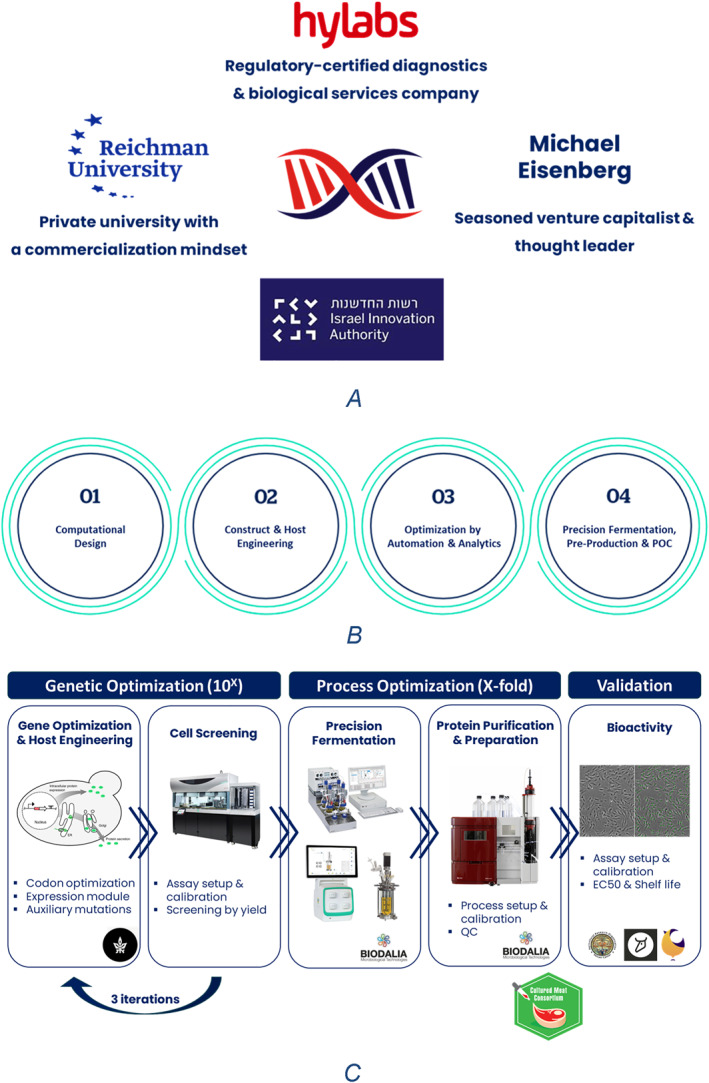
Alagene, first Israeli Bio‐Foundry. (a) A Unique Venture Facilitated by a Governmental Fund to Ramp Up SynBio in Israel. (b) An end‐to‐end solution: Four modules process for SynBio ideas to industrial Proof of Concepts (POCs). (c) Schematic description of the development of a cost‐efficient growth factor production system for the cultured meat industry.

Alagene's vision is to become a SynBio powerhouse and to nourish the international SynBio ecosystem by providing cutting‐edge SynBio R&D services; alleviating technological, operational, and knowhow‐related barriers; making frontline practices associated with computational design and engineering of genes and organisms; and making high‐throughput constructing and screening, analytics, precision fermentation, and prototyping accessible for all across the academy and industry (See Figure 2). The allocated budget also enabled the company to bring back SynBio experts from the USA and to hire brilliant local leaders.

Alagene Ltd. is composed of a seasoned and highly skilled scientific team, equipped with state‐of‐the‐art instrumentation, to bring to fulfilment ambitious SynBio development processes such as optimization of catalysts, heterologous production of biomolecules, design of novel genetic programs and reporter systems, and many more applications.

Alagene offers an inclusive development pipeline that follows the Design‐Build‐Test‐Learn paradigm and is divided into four operation modules. The Computational Design module (module 1 in Figure 2. B) employs data science, structural biology, and bioinformatics to generate data and AI‐driven predictions aiming to direct the biological system towards its applicative designation. The Construct and Host Engineering module (module 2) utilises genetic engineering procedures and automation to streamline the construction of genetic libraries, composed of variants generated by rational‐design or randomisation approaches. The Optimization by Automation and Analytics module (module 3) makes use of automation to screen numerous variants in a high throughput manner in a fast, accurate, and cost‐efficient manner. Sophisticated analytic methods determine analyte structure and quantity. The Precision Fermentation, Pre‐Production and proof of concept (POC) module (module 4) is taking the genetic optimization effort the extra mile towards commercialisation by complementing the superior genetics with process development, for optimising its performance in applications such as bioproduction or implementation of the engineered system in a given use case. This development process ultimately supports SynBio product development from the very early ideation phase up to the completion of a POC.

Alagene collaborates with academic groups, industrial ventures, and national institutes in various projects, associated with multiple industries, including pharma, cosmetics, ag‐tech, cleantech, food‐tech, materials, energy, and defence. One example can be found in Figure 2c.

**FIGURE 3:**
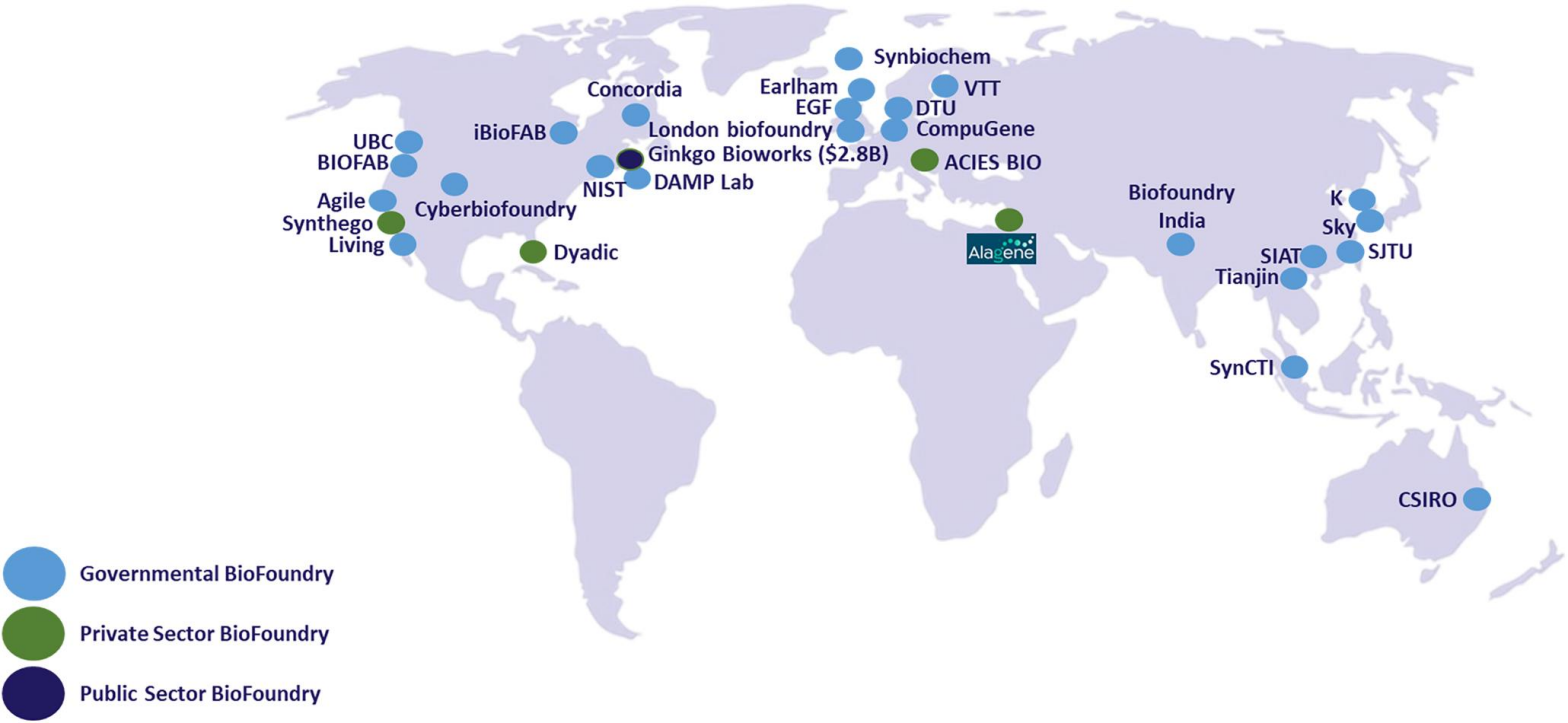
Global landscape of bio‐foundries.

Engineering life for the benefit of humanity, Alagene is quickly becoming a leader in the global market, side by side with other bio‐foundries, some of which are mentioned in Figure 3. Alagene is a home for innovation and R&D in SynBio, serving as a one‐stop—shop for knowledge, methods, and instrumentation to enable customers in Israel and worldwide to get to their POC.

### 5.2 Application‐oriented government initiatives

Some of the most interesting applications in the synthetic biology field have started due to government initiatives, mostly in academia but also in the start‐up field. Many of the applications are in the food‐tech, aggrotech, and medical domains. For example, Imagindairy Ltd., a startup that was founded based on Professor Tuller's technology, is focused on natural precision fermentation to create milk proteins that are not based on animal‐based dairy. Piotrix EPius developed a treatment based on the toxoplasma parasite engineered to create and secrete the MECP2 protein within neurons that will replace the defective protein in Radiation Therapy (RT) patients. All those companies were supported by the IIA, which sometimes also makes connections with academia and other partners.

Other relevant companies that have entered the field include Wild Biotech, which employs an end‐to‐end process that goes from field exploration and discovery to drug development and clinical trials. TargetGene was founded in 2012 by Drs. Dan Weinthal and Yoel Shiboleth. Using their 2011 invention of RNA‐guided nucleases for genome editing, they are now developing a new genome editing platform. In the agricultural field, one can identify mature Israeli companies such as Hazera, Rahan, and BeterSeeds.

The IIA is lately encouraging government‐funded incubators to add Bio‐synthetics companies to their portfolio. For example, the Kitchen incubator has included the company Mitologic, which utilises Dr. Nissim's cancer immunotherapy technology. MeatoLogic is a cultivated meat company that employs advanced SynBio tools to develop cells that can produce autonomously, with minimal interventions, cost‐efficient cultivated meat products. Bio Circuit, a company in the Futurex incubator portfolio, is a startup that developed a complex genetic circuit for the treatment of colorectal and breast cancers.

### 5.3 Local consortia

This Israeli incentive consortium programme provides grants for R&D collaboration as part of a group of industrial companies and research institutions developing technologies together. The various consortiums in the programme enable a long‐term engagement in SynBio R&D and create a supportive work environment. For companies from the industry, this is an opportunity to collaborate with other companies and research groups from academia that help develop groundbreaking technology. For research institutions, collaboration with industry advances the commercialisation of products based on academic research, as well as an understanding of market needs. The consortium has operated for 3 years.

The consortium's goal is to support the development of generic technologies in important fields in the global market, in which Israeli industry has, or may have a competitive advantage. Since this incentive programme supports the funding of infrastructure technologies, it allows the distribution of knowledge and cooperation between companies operating in the same field, which may be difficult to achieve otherwise.

Below are three examples of relevant consortia funded by the IIA.

#### 5.3.1 The cultured meat consortium

The IIA has approved the establishment of the Cultivated Meat Consortium, one of the biggest in the world. Together with the business sector and academia, 66M NIS will be invested in the consortium over 3 years, half of the sum by the government. The consortium aims to develop innovative production methods on an industrial and efficient scale, for a sector still in its infancy, to provide Israel's cultivated meat industry with a competitive advantage in international markets. The consortium, which is led by Israeli food giant Tnuva, encompasses 14 companies and 10 academic laboratories. Consortium members also include Aleph Farms, Super Meat, Seevix, Alagene, the Hebrew University of Jerusalem, Reichman University, and TAU. The consortium tackles interdisciplinary topics ranging from the shelf life of products, regulation, new foods, use of existing food production lines for ingredients, significantly reducing production costs, and more. Four working groups are operating, focussing on developing technologies for cell growth for muscle and fat, bioreactor technologies, and developing production, scaffold technologies, and growth technologies and their components. Tnuva's chief innovation officer and corporate venturing officer, Shay Cohen, serves as chairman of the consortium.

#### 5.3.2 The CRISPRIL consortium

The CRISPRIL consortium was established to address the following challenges of the Clustered Regularly Interspaced Short Palindromic Repeats (CRISPR) technology:

(i) Accidental “off‐target” modification,
(ii) Low “on‐target” efficiency requiring repeated trials,
(iii) Inaccurate tools for validating the planned modifications.

This was achieved by developing an artificial intelligence‐based system, “GOGENOME,” providing improved genome‐editing workflows with high accuracy and efficacy. Clustered Regularly Interspaced Short Palindromic Repeats has been a groundbreaking and leading technology in the biotech and health industry in recent years. The ability to generate precise genomic modifications in a relatively simple and efficient manner revolutionised the ability of scientists to study various types of diseases, develop improved crops and livestock, and understand genetic processes. Harnessing CRISPR in medicine and agriculture will ultimately lead to the development of safer and cheaper solutions, worth billions of dollars, and address some of the critical challenges of our time. Although CRISPR‐Cas is considered convenient for specific targeting of the desired trait, it still faces several challenges, preventing more extensive use and products in the markets.

#### 5.3.3 The Israeli generic bio‐chip technology R&D consortium

The Bio‐Chip consortium consists of 7 industrial companies (Tower SC, CEVA, PLSense QuLab medical, Cardiokol, SensoMedical, Israel Aerospace Industry) and 12 research groups from 4 different academic institutes (The Technion IIT, BIU, BGU and TAU). The main goal of the consortium is to develop and bring to market innovative‐generic Bio‐chips technologies that will be available for the emerging Israeli Bio‐Medical sector and will bridge the currently existing gap in the industry. The consortium develops various biosensors and supportive technologies with an emphasis on innovative low‐power circuits, chip manufacturing technologies, microfluidics, optical applications, and AI capabilities needed for bio‐chip‐based systems. The technologies will be demonstrated in metabolic sensing, innovative heart‐arrhythmia detection, analog biosensors, EEG, SynBio, and more.

## 6 EDUCATION & TRAINING

As with any other ecosystem, education, and training are crucial for SynBio's success. SynBio was first taught at WIS, Technion, BGU, and TAU in academic courses for graduate students. Recently, other universities and colleges have created additional courses and planned programs as well. The first subsection below is about local‐focused programs supporting industrial growth. The second subsection is about younger generations taking courses in middle and high schools around the country.

### 6.1 Focused advanced programs

Some advanced programs have been developed to take skilled individuals and introduce them to the SynBio field. One such programme, called SmashingDNA is aimed at ‘hackers’ and reverse engineers from elite army units that are interested in biology. Another interesting initiative is called SpearHealth, which addresses top tech and business talents to become leaders in the field of SynBio HealthTech.

A few universities in Israel including the Technion, BGU, and Tel‐Aviv University are involved in the international genetically engineered machine (iGEM) competition, a global SynBio competition. The competition was founded in 2004 for undergraduate students at MIT but has since expanded to thousands of participants from all over the world: high school students, students, entrepreneurs, and community labs (e.g. from the past few years see https://2020.igem.org/Team:TAU_Israel and https://2022.igem.wiki/tau‐israel/).

In addition to these courses and competitions, a novel graduate programme is to be launched at the HIT. This is the first Israeli programme for bio‐convergence and SynBio. It will emphasise the integration of high‐end academic research with industrial and governmental needs. We are sure that many other courses and programs are soon to be developed as more PIs relocate back to Israel and as the local industry continues its current growth. Holon Institute of Technology faculty currently consists of a few relevant PIs in bioengineering, SynBio, and other relevant areas. Bioengineering is led by Dr. Ronen Sosnik The bio‐convergence centre at HIT is currently led by Dr. Yuval Dorfan mentioned above in the academic section.

### 6.2 School education

Besides these professional courses and programs, there are interesting initiatives for middle and high school students. The education system in Israel does not teach the field of SynBio yet. Therefore, any activity in this field will be defined as a personal or organizational initiative. As of 2023, the exposure of high school students to the field of SynBio is done mainly in two ways: participation in the iGEM competition or computational biology studies in an academic track.

In 2015, a team of high school students from Israel participated for the first time in the iGEM competition. Dr. Magi Mualem, a dominant high‐school teacher led a group of biotechnology students from the Danziger School in Kiryat Shmona to the iGEM competition. With the professional assistance of Dr. Itamar Yadid from Tel Hai College, the students created a homemade kit for identifying gluten in food. Before the trip to the competition, Dr. Mualem produced the first SynBio conference for teenagers. The conference was attended by over 400 high school students from Israel as well as leading researchers and students from the SynBio field. In 2016, Dr. Moalem led another group of students to the iGEM competition. This time, Professor Danny Berkowitz and Mrs. Gita Reinitz assisted in the professional consultation. The students presented a system, based on CRISPR, that performs genetic editing of the sense of taste. The students were accompanied by the Technion team as part of the “Scientists of the Future” project.

Additional exposure to the SynBio field is carried out as part of the “Chimera” programme. The Chimera programme was founded in Jerusalem in 2022 by the Jerusalem Municipality with the assistance of the Hebrew University and the Aleph Fund. The programme is intended for gifted students from 10th grade. The purpose of the programme is to expose students to the SynBio field in both theoretical and practical ways.

## 7 CONCLUSIONS AND FUTURE PERSPECTIVE

As for many previously developed technologies, Israel identified the importance of SynBio relatively early. The build‐up of an ecosystem has started based on local private initiatives. However, to make a significant impact, a global public policy is required. Israeli spirit as a start‐up nation combined with many other academic and industrial strengths are the main reasons for the early signs of success. The young SynBio ecosystem in Israel is expected to grow exponentially in the coming years thanks to a few elements that will work harmonically together: academia, industry, government, and the education and training systems. Israel's SynBio ecosystem is highly connected to the global ecosystem with the vision to improve human life on Earth. The way the state of Israel initiated this ecosystem is different compared to previous technologies such as cyber and communication. For SynBio the IIA identified it as a growth engine in its very early steps and invested money in infrastructure and mechanisms that glue all stakeholders' efforts together. It enabled us to close many gaps quickly and enable new ideas to become new companies. We think this could be learnt as a case study both for other countries interested in the SynBio area and even for future technologies that are still within their basic research stage.

## AUTHOR CONTRIBUTIONS

**Yuval Dorfan**: Conceptualisation; Data curation; Funding acquisition; Investigation; Methodology; Project administration; Resources; Supervision; Validation; Visualisation; Writing – original draft; Writing – review & editing. **Aviv Zeevi**: Conceptualisation; Data curation; Investigation; Supervision; Writing – original draft. **Gita Reinitz**: Conceptualisation; Investigation; Writing – original draft. **Magi Mualem**: Conceptualisation; Writing – original draft. **Yosi Shacham‐Diamand**: Conceptualisation; Investigation; Project administration; Supervision; Validation; Writing – original draft; Writing – review & editing.

## CONFLICT OF INTEREST STATEMENT

The authors declare that they have no known competing financial interests or personal relationships that could have appeared to influence the work reported in this paper.

## Data Availability

The data that support the findings of this study are available on request from the corresponding author. The data are not publicly available due to privacy or ethical restrictions.
